# 

**Published:** 1890-01

**Authors:** 


					To Readers and Correspondents.
Communications intended for publication, and exchange journals
and papers, should be addressed to the Editor of The American
Journal of Dental Science, No. 845 N. Eutaw St. Baltimore, Md.
All letters relating to the business of the Journal, or containing cash
remittances, to be directed to Messrs. Snowden & Cowman. No. 9 W.
Fayette Street, Baltimore, Md. Articles ior publication should be re-
ceived by the Editor at least two weeks previous to the first of the
month on which the number in which it is designed for them to appear,
is issued.
The American Journal of Dental Science will contain fifty
pages of reading matter in each number, making a volume
of about Six Hundred pages,
EXCLUSIVE OF ADVERTISEMENTS.

				

